# Chemogenetics with PSAM^4^-GlyR decreases excitability and epileptiform activity in epileptic hippocampus

**DOI:** 10.1038/s41434-024-00493-7

**Published:** 2024-10-25

**Authors:** Ana Gonzalez-Ramos, Fredrik Berglind, Jan Kudláček, Elza R. Rocha, Esbjörn Melin, Ana M. Sebastião, Cláudia A. Valente, Marco Ledri, My Andersson, Merab Kokaia

**Affiliations:** 1 Epilepsy Center, Department of Clinical Sciences, Lund University Hospital, Lund, Sweden; 2 Instituto de Farmacologia e Neurociências, Faculdade de Medicina, Universidade de Lisboa, Lisboa, Portugal; 3 Instituto de Medicina Molecular João Lobo Antunes, Universidade de Lisboa, Lisboa, Portugal; 4 Present Address: Stanley Center for Psychiatric Research, Broad Institute of MIT and Harvard, Cambridge, MA USA; 5 Present Address: Department of Physiology, Second Faculty of Medicine, Charles University, Prague, Czech Republic

**Keywords:** Neuroscience, Neurological disorders

## Abstract

Despite the availability of new drugs on the clinics in recent years, drug-resistant epilepsy remains an unresolved challenge for healthcare, and one-third of epilepsy patients remain refractory to anti-seizure medications. Gene therapy in experimental models has emerged as effective treatment targeting specific neuronal populations in the epileptogenic focus. When combined with an external chemical activator using chemogenetics, it also becomes an “on-demand” treatment. Here, we evaluate a targeted and specific chemogenetic therapy, the PSAM/PSEM system, which holds promise as a potential candidate for clinical application in treating drug-resistant epilepsy. We show that the inert ligand uPSEM^817^, which selectively activates the chloride-permeable channel PSAM^4^-GlyR, effectively reduces the number of depolarization-induced action potentials in vitro. This effect is likely due to the shunting of depolarizing currents, as evidenced by decreased membrane resistance in these cells. In organotypic slices, uPSEM^817^ decreased the number of bursts and peak amplitude of events of spontaneous epileptiform activity. Although administration of uPSEM^817^ in vivo did not significantly alter electrographic seizures in a male mouse model of temporal lobe epilepsy, it did demonstrate a strong trend toward reducing the frequency of interictal epileptiform discharges. These findings indicate that PSAM^4^-GlyR-based chemogenetics holds potential as an anti-seizure strategy, although further refinement is necessary to enhance its efficacy.

## Introduction

Epilepsy is the fourth most common neurological disorder affecting over 50 million people worldwide [1]. Despite the existence of symptomatic pharmacological treatments known as antiseizure medications (ASMs) [2–5], there are no preventive or disease-modifying treatments available in clinical settings [6]. Importantly, 30–40% of the patients do not respond to ASM, and thus drug-resistant epilepsy constitutes a significant burden in healthcare [7]. For a small subset of drug-resistant patients, fewer than 5% [8–10], surgical resection of the epileptogenic focus can be effective. However, up to 40% of patients undergoing such surgeries experience early or late surgical failures [11]. Consequently, there is a critical need for effective disease-modifying and/or antiseizure treatments to manage seizures in this drug-resistant group.

In humans, the most common form of focal epilepsy among drug-resistant patients is temporal lobe epilepsy (TLE) with hippocampal sclerosis (HS), a well-characterized epileptic syndrome originating from the mesial temporal lobe, featuring focal spontaneous recurrent seizures (SRSs) that may generalize [12, 13]. Some of the most characteristic histopathological hallmarks of the disorder are severe neuronal loss predominantly in the *cornu ammonis* 1 (CA1) area of the hippocampus, accompanied by reactive gliosis [14], dispersion of the granule cells (GCs) of the dentate gyrus (DG) [14] and mossy fiber sprouting [15]. Moreover, degeneration of hippocampal gamma-aminobutyric acid (GABA)-ergic interneurons has also been described which encompasses a decreased synaptic inhibition of the GCs [16–21]. Observations in animal models point out that GABAergic neuronal loss may play an important role in promoting the epileptic state [22, 23]. This was further supported by studies showing seizure reduction after the replacement of the missing GABAergic neurons by an exogenous cell source [24–28] or by activation of the surviving endogenous interneurons [29].

Previous studies have shown promising results by modulating the activity and/or survival of certain neurons in the epileptogenic focus by overexpressing or dampening the expression of certain genes using gene therapy [30–33]. In these cases, the gene of interest is delivered by the injection of viral vectors containing the gene sequence, which will then be expressed in specific cell types depending upon the promoter and the tropism of the viral vector serotype used [34]. Moreover, the viral vectors can also be engineered to carry genes encoding for proteins that are externally regulated by drugs or light, known as chemogenetics or optogenetics respectively. These have also been utilized and allow adjustment of the therapeutic effects in terms of dose and time of action. Chemogenetics enables cell-type-specific modulation, either reducing neuronal excitability or enhancing neuronal activity, ultimately leading to seizure inhibition [35–40]. Previous studies have used Designer Receptors Exclusively Activated by Designer Drugs (DREADD) derived from G-protein-coupled receptors activated by a neutral designer drug known as clozapine N-oxide (CNO). The advantage of DREADDs over optogenetics is that this approach is less invasive, not needing optical fiber implantation, and leads to longer effects due to activator pharmacokinetics. Recent research has revealed that CNO has limited capacity to cross the blood-brain barrier (BBB). Consequently, its primary effect on DREADDs expressed in the brain is likely due to its back conversion to clozapine [41], a molecule with complex pharmacology that crosses the BBB and interacts with multiple receptors in the brain even at low concentrations [42]. A way to overcome these limitations in clinical translation is to use another chemogenetic tool based on synthetic ligand-gated ion channels (LGIC) which has an activator already approved for clinical use in other pathologies. Numerous studies on brain function have employed anion-permeable ligand-gated ion channels (LGICs), known as pharmacologically selective actuator modules (PSAM), to inhibit neurons by shunting excitatory currents and hyperpolarizing the membrane, depending on the transmembrane chloride gradient [43, 44]. However, as far as we know, PSAM has not yet been utilized to manage seizures in epilepsy models.

In this study, we have investigated the potential anti-seizure effect of PSAM^4^-GlyR, a relatively new inhibitory ligand-gated ion channel (LGIC). PSAM^4^-GlyR is a chimeric protein composed of a modified ligand-binding domain from the α7 nicotinic acetylcholine receptor (α7-nACh) fused to the anion-permeable ion-pore domain of a glycine receptor (GlyR) [43, 44]. The modifications in the ligand-binding domain include three specific mutations—α7L131G, Q139L, and Y217F—which enhance sensitivity to the activator uPSEM^817^ as well as to varenicline, an FDA-approved partial agonist of the α4β2-nAChR used for smoking cessation. To evaluate the anti-seizure potential of PSAM4-GlyR, we employed the intrahippocampal kainic acid (IHKA) mouse model of chronic temporal lobe epilepsy (TLE). We generated recombinant adeno-associated viral vectors (AAVs) using Ca^2+^/calmodulin-dependent kinase II alpha (CaMKIIα) promoter to selectively target pyramidal cells (Pyr) and GCs in the hippocampus. We then evaluated the effect of the pharmacologically selective effector molecules (uPSEM^817^) on these cells in acute brain slices from chronic epileptic male mice, observing a shunting inhibition of the cell responses to depolarizing currents. In an ex vivo epilepsy model using entorhinal cortex-hippocampus organotypic slices from rats, uPSEM^817^ reduced bursting activity. Finally, in vivo-EEG-video monitoring of epileptic male mice revealed that while uPSEM^817^ tended to reduce the rate of interictal epileptiform discharges (IEDs), it did not significantly inhibit electrographic seizures (ESs). These findings suggest that the PSAM^4^-GlyR/uPSEM^817^ system has therapeutic potential to reduce epileptiform activity, although further optimization is needed for effective in vivo anti-seizure responses.

## Results

### PSAM^4^-GlyR expression and histopathology after the intrahippocampal kainic acid injection

**Fig. 1:**
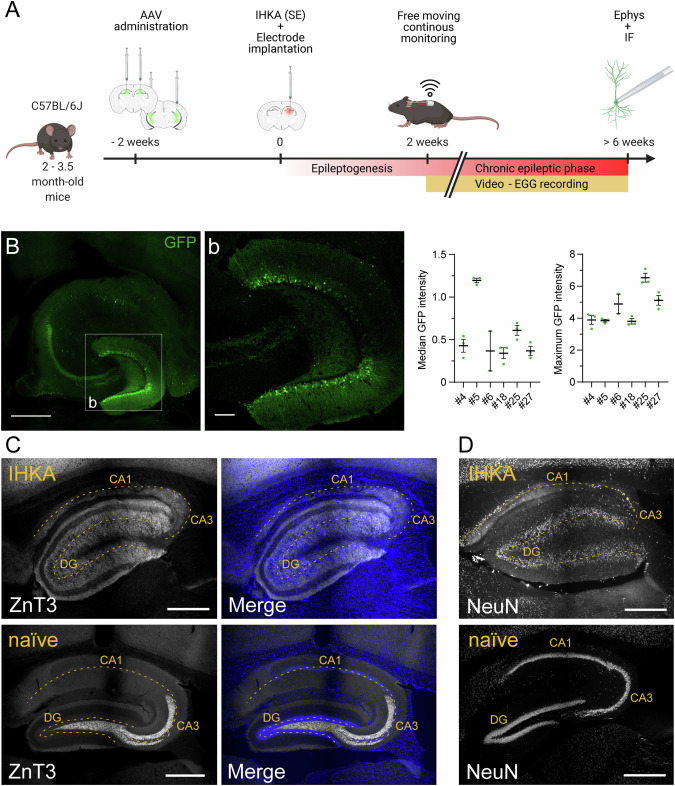
Experimental design, PSAM^4^-GlyR expression, and histopathology after the IHKA injection. **A** Schematics of the study timeline. IHKA intrahippocampal kainic acid; SE status epilepticus; Ephys electrophysiology; IF immunofluorescence; EEG electroencephalogram. **B** Maximum intensity projection of immunofluorescence showing PSAM^4^-GlyR-IRES-eGFP expression across the hippocampus in a horizontal section. **b** Magnification of the dentate gyrus on a z-stack plane. On the right, quantification of median and maximum GFP+ fluorescence intensity of the DG area in the different PSAM^4^-GlyR animals used in the analysis. **C** Immunofluorescence for mossy fiber sprouting, using antibodies against ZnT3, and merged with the nuclear staining DAPI in naïve (bottom) and IHKA (top) mice (indicated in yellow). **D** Immunofluorescence for neuronal nuclei, NeuN, in naïve (bottom) and IHKA (top) mice. Images correspond to the surrounding area of the IHKA depot and electrode location in the right dorsal hippocampus, sagittal sections. Tissue was collected following the completion of in vivo recordings, using a vibratome to prepare slices for acute electrophysiology experiments. The dashed yellow line in (**C**, **D**) represents the cell body layers highlighting the anatomical structure of the hippocampus. Scale bar: 500 µm (**B**–**D**) and 100 µm (**b**).

To assess the antiseizure potential of PSAM^4^-GlyR activation, we first injected the AAV8-CaMKIIα- PSAM^4^-GlyR-IRES-eGFP vector into both hippocampi of 21 male C57BL/6J mice (Fig. 1A). Seven male C57BL/6J mice were injected with control viral vectors containing AAV8-CaMKIIα-eGFP. Viral expression was mainly restricted to the dentate GCs, although some Pyr in CA3 and CA1 were also GFP positive (Fig. 1B). Similar levels of expression were observed in all animals (Fig. 1B). The predominant expression of the transgene in the DG was considered potentially adequate to meet the objectives of the study, given the established role of DG in the generation and propagation of seizure activity [45–47]. Additionally, prior research indicates a reduction in the number of GABAergic inhibitory interneurons in the DG of patients with epilepsy, further emphasizing the importance of targeting this region [21].

Two weeks after viral injections, status epilepticus (SE) was induced in the animals by performing KA injections into the right dorsal hippocampus. During the same surgical procedure, an electrode connected to a wireless transmitter was also implanted at the specified coordinates, as shown in Fig. 1A. From the initial cohort of the animals, we observed a mortality rate of 35.7% during SE induction (10 died out of 28 mice). This is likely due to simultaneous IHKA injections and electrode implantation, which may have enhanced kainic acid spread, resulting in a more severe epileptic phenotype. Following status epilepticus (SE), a period of ~2 weeks was allowed for epileptogenesis to occur, leading to the subsequent development of SRS. After this 2-week period, continuous video-EEG recordings were initiated, marking the beginning of the chronic phase of epilepsy characterized by SRS (Fig. 1A). We observed IEDs in all animals (5 animals were discarded due to bad recording signal). The IEDs occurred at a considerably low rate in mice where seizures were not detected and consequently, such animals were discarded from the study (*n* = 4). The cut-off value for exclusion was ≤10 IEDs/hour, while on average IED rate in the saline (negative control) treated PSAM^4^-GlyR virus-injected animals was 147 ± 435 events/hour, and in GFP-only virus-injected animals it was 63 ± 150 events/hour (median ± interquartile range). The rate and amplitude of IEDs during baseline recording did not differ significantly between experimental groups (Ctrl / GFP-only vs. PSAM^4^-GlyR; *p* = 0.89; Supplementary Fig. 1). All mice with a high rate of IEDs (>10 IEDs/hour, *n* = 9) exhibited behavioral generalized seizures (PSAM^4^-GlyR *n* = 7, and control *n* = 2). Focal nonconvulsive electrographic seizures (ESs) were detected in four mice (44.4%).

To verify that our IHKA-treated mice exhibited the typical histopathological changes associated with TLE, such as structural network reorganizations and GC layer dispersion, we conducted an immunohistochemical analysis. This included staining for mossy fiber sprouting using zinc transporter 3 (ZnT3) and assessing neuronal body localization with neuronal nuclear antigen (NeuN) staining. Representative images from stainings on IHKA and naïve mice are shown in Fig. 1C illustrating substantial mossy fiber sprouting in the inner molecular layer of the DG in IHKA mice compared to naïve ones (Fig. 1C). Moreover, dispersion of the GC layer was observed in IHKA mice, as well as severe neuronal loss in the CA1 (Fig. 1D).

### Activation of PSAM^4^-GlyR-transduced neurons by uPSEM^817^ decreases input resistance and the rate of action potentials induced by depolarizing currents

**Fig. 2:**
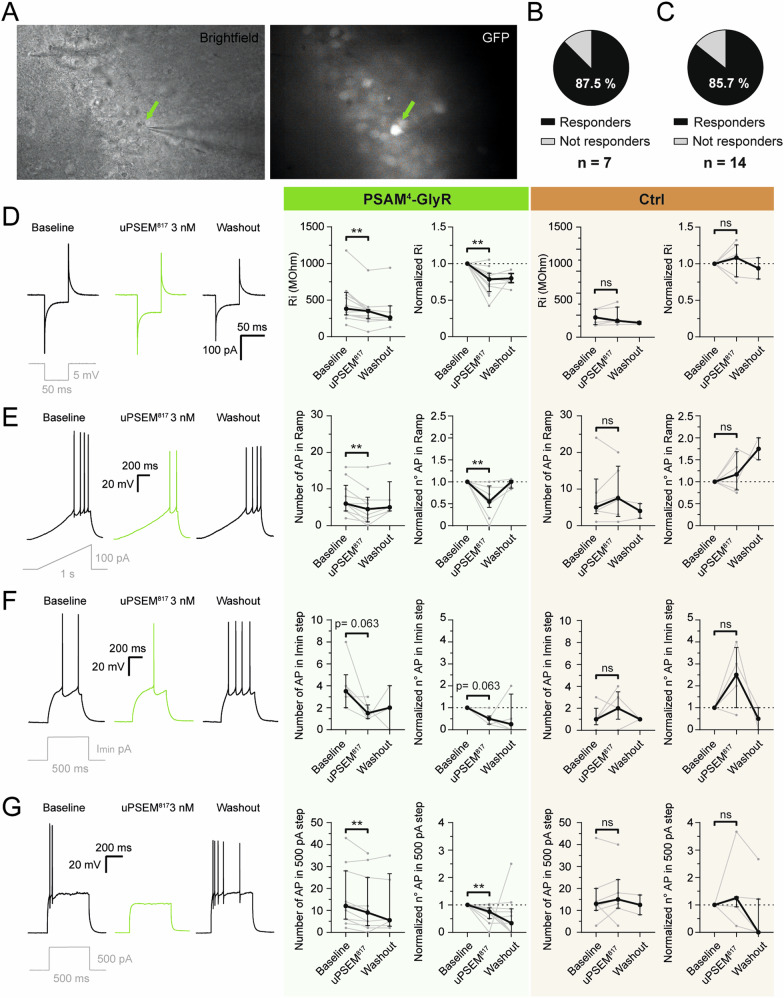
Effect of uPSEM^817^ on intrinsic electrophysiological properties of the cells expressing PSAM^4^-GlyR. **A** Whole-cell patch-clamp recording of a GFP+ cell (green arrow). The Infrared differential interference contrast (IR-DIC) image on the left and fluorescent 470 nm light visualization on the right. Pie chart illustration of the percentage of PSAM^4^-GlyR treated animals ((**B**), *n* = 7), and all measured CaMKIIα-PSAM^4^-GlyR-GFP+ cells from those animals ((**C**), *n* = 14), undergoing changes in intrinsic properties when uPSEM^817^ was washed in. **D** Test pulse response in the different treatments, green indicating uPSEM^817^ application in PSAM^4^-GlyR-GFP+ cells. From left to right, Ri measurement and normalized values in CaMKIIα-PSAM^4^-GlyR-eGFP+ cells (green) and CaMKIIα-eGFP+ cells (Ctrl, orange) before, during, and after uPSEM^817^ application. **E** Differential cell response to 0–100 pA ramps of depolarizing current in different treatments, green indicating uPSEM^817^ application in PSAM^4^-GlyR-GFP+ cells. From left to right, number of APs in response to ramps of depolarizing current and normalized values in CaMKIIα-PSAM^4^-GlyR-eGFP+ cells (green) and CaMKIIα-eGFP+ cells (Ctrl, orange) before, during, and after uPSEM^817^ application. **F** Differential cell response to the lowest depolarizing current pulses for AP induction abbreviated as Imin (in this case 80 pA) in the different treatments, green indicating uPSEM^817^ application in PSAM^4^-GlyR-GFP+ cells. From left to right, number of APs in response to Imin current pulses and normalized values in CaMKIIα-PSAM^4^-GlyR-eGFP+ cells (green) and CaMKIIα-eGFP+ cells (Ctrl, orange) before, during, and after uPSEM^817^ application. **G** Differential cell response to 500 pA depolarizing current pulses in the different treatments, green indicating uPSEM^817^ application in PSAM^4^-GlyR-GFP+ cells. From left to right, number of APs in response to 500 pA pulses and normalized values in CaMKIIα-PSAM^4^-GlyR-eGFP+ cells (green) and CaMKIIα-eGFP+ cells (Ctrl, orange) before, during, and after uPSEM^817^ application. Ri, input resistance; Imin, minimum current pulse step to trigger an AP. Animals injected with AAV8-CaMKIIα-PSAM^4^-GlyR-IRES-eGFP, *n* = 7 (total cells *n* = 14); control/GFP-only animals injected with AAV8- CaMKIIα-eGFP, *n* = 3 (total cells *n* = 7). Cells used in the analysis as responders *n* = 12. Median ± interquartile range. Wilcoxon paired test for comparison of the PSEM effect to the Baseline. **, *p* < 0.01.

**Fig. 3:**
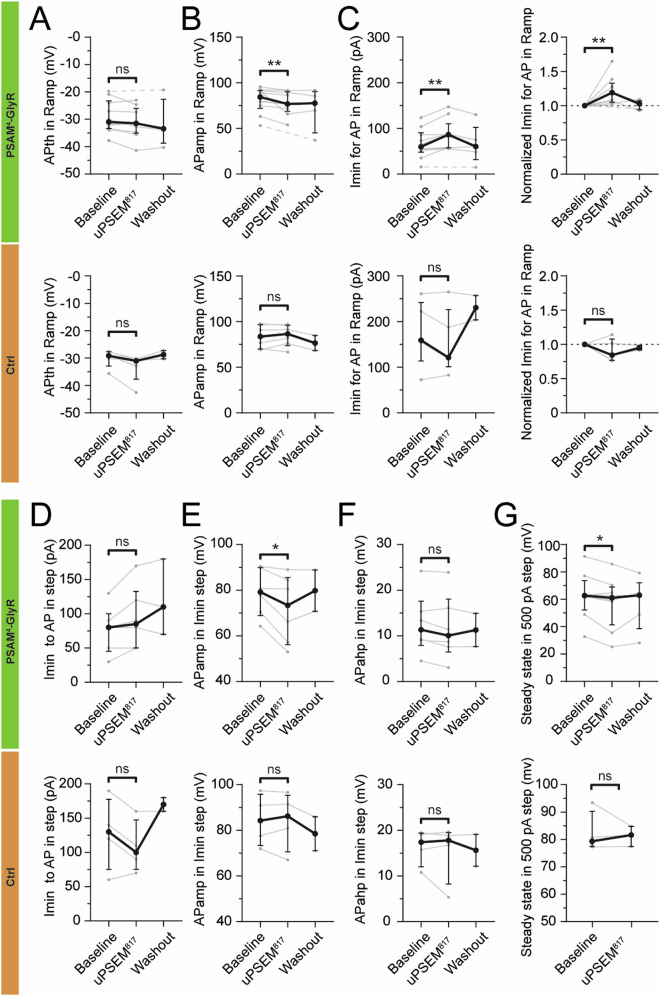
The effect of uPSEM^817^ on PSAM^4^-GlyR causes alterations in some evoked action potential properties. Whole-cell patch-clamp recording of CaMKIIα-PSAM^4^-GlyR-eGFP+ cells (green) and control GFP-only cells (Ctrl, orange). Measurements in depolarizing ramp currents of the APth (**A**), APamp (**B**), and Imin for the first AP ((**C**), left). Normalized values for Imin for the first AP ((**C**), right). The minimum current needed to trigger APs in depolarizing step currents (**D**). Measurements in Imin depolarizing step for APamp (**E**) and APahp (**F**). Steady-state current amplitude in 500 pA step (**G**). AP, action potential; Imin, minimum current to trigger an AP in step currents; APth AP threshold; APamp AP amplitude; APahp AP afterhyperpolarization amplitude. The dashed line indicates that the cell did not generate an AP during the uPSEM^817^ effect, thereby there is no value in the analysis for that cell and the line connects the value from the baseline directly to the wash-out. CaMKIIα-PSAM^4^-GlyR-eGFP+ cells: *n* = 12, CaMKIIα-eGFP+ cells: *n* = 7. Median ± interquartile range. Wilcoxon paired test for comparison of the PSEM effect to the Baseline. *, *p* < 0.05; **, *p* < 0.01.

We first investigated the effect of PSAM^4^-GlyR activation by uPSEM^817^ at the single-cell level in acute brain slices in vitro. PSAM^4^-GlyR consists of a ligand-binding domain of the α7 nicotinic acetylcholine receptor (α7-nACh) fused to the chloride-conducting pore of a glycine receptor (GlyR) [43, 44]. Therefore, the outcome of PSAM^4^-GlyR activation will depend on the concentration of chloride ions inside vs. outside the cells in the brain tissue. Chloride homeostasis in TLE is a controversial topic [48], some studies suggest depolarizing effects of GABA_A_ receptor activation in tissue from patients with epilepsy [49], although others show hyperpolarization by GABA [50, 51]. With that premise, we performed whole-cell patch-clamp recordings on GFP+ cells (Fig. 2A) from both PSAM^4^-GlyR (*n* = 14 cells) and GFP-only animals (*n* = 7 cells). To assess the effect of uPSEM^817^, for each cell recording in the acute slices, a baseline period was recorded prior to the start of perfusion with uPSEM^817^ at a final concentration of 3 nM into the recording bath, followed by a wash-out period (see Methods). From all the PSAM^4^-GlyR cells recorded, only 2 of them (coming from the same animal) were considered as non-responders, meaning that none of the parameters analyzed were changed by application of uPSEM^817^ (Fig. 2B, C). Among the responders, uPSEM^817^ decreased input resistance (Ri) most likely due to the opening of PSAM^4^-GlyR channels (−21.53% [range −13.1 to −38.3]; *p* = 0.0015; Fig. 2D, green), while it did not affect Ri in control GFP-only cells (+8.2% [range −17.8 to +25.7]; *p* = 0.5781; Fig. 2D, orange). Note that all data given here and in the next section (Figs. 2 and 3, respectively) are summarized in Supplementary Table 1. Chloride flow through the PSAM^4^-GlyR channels after activation by uPSEM^817^ resulted in a decreased number of action potentials (APs) triggered by depolarizing currents, both as a ramp (Fig. 2E, green) and as 500 pA steps (Fig. 2G, green). For the depolarizing ramp current, uPSEM^817^ decreased the number of APs by 45% [range 9.37–58.33] (*p* = 0.0078) in the PSAM^4^-GlyR cells (Fig. 2E, green), while in the control GFP-only ones there were no differences observed (increase by 16.7% [range −18.75 to +68.8]; *p* = 0.3125; Fig. 2E, orange). No statistically significant differences were observed for the number of APs during Imin, the lowest current step for AP induction, although there was a clear trend of reduction (decrease by 50% [range 37.5–75]; *p* = 0.0625; Fig. 2F, green). At the 500 pA step however, there was a statistically significant reduction of the number of APs compared to the baseline in the PSAM^4^-GlyR cells (decrease by 25% [range 10.71–50]; *p* = 0.0039; Fig. 2G, green), but not in the GFP-only cells (with lowest current step increase by 150% [range 0–275]; *p* = 0.25; Fig. 2F, orange; and with 500 pA step increase by 25% [range −7 to +30]; *p* = 0.2969; Fig. 2G, orange).

The decrease in the number of APs in response to depolarizing ramp and step currents was most likely due to a shunting effect of PSAM^4^-GlyR opening [52], which would require higher amplitude current pulses for AP induction. In particular, the minimum current needed in the depolarizing ramp currents during the effect of uPSEM^817^ was 19% [range 4.8–32.9] larger than before uPSEM^817^ application in PSAM^4^-GlyR expressing cells (*p* = 0.0039; Fig. 3C, top green), whereas it was not altered in the GFP-only cells (*p* = 0.3125; Fig. 3C, bottom, orange).

More detailed analysis revealed that the AP threshold was unaltered during uPSEM^817^ application (*p* = 0.164; Fig. 3A, D), nor was the afterhyperpolarization amplitude at the lowest step currents inducing APs (*p* = 0.156; Fig. 3F). However, AP amplitude was decreased in PSAM^4^-GlyR expressing cells after the application of uPSEM^817^ both at depolarizing ramp currents (*p* = 0.0039; Fig. 3B, top green) and lowest step currents (*p* = 0.0313; Fig. 3E, top green), but not in GFP-only cells (*p* = 0.563 and *p* = 0.875 respectively; Fig. 3B and E, bottom orange). Similarly, the steady-state amplitude for 500 pA step currents was reduced during the uPSEM^817^ effect in PSAM^4^-GlyR expressing cells (*p* = 0.0234; Fig. 3G, top green), but not in GFP-only ones (*p* > 0.999; Fig. 3G, bottom orange). Decreased AP amplitudes and steady-state amplitude could be explained by the shunting effect on AP sodium currents exerted by the activation of exogenous chloride-permeable PSAM^4^-GlyR channels.

### Decreased epileptiform activity in organotypic slices by uPSEM^817^ activation of PSAM^4^-GlyR

**Fig. 4:**
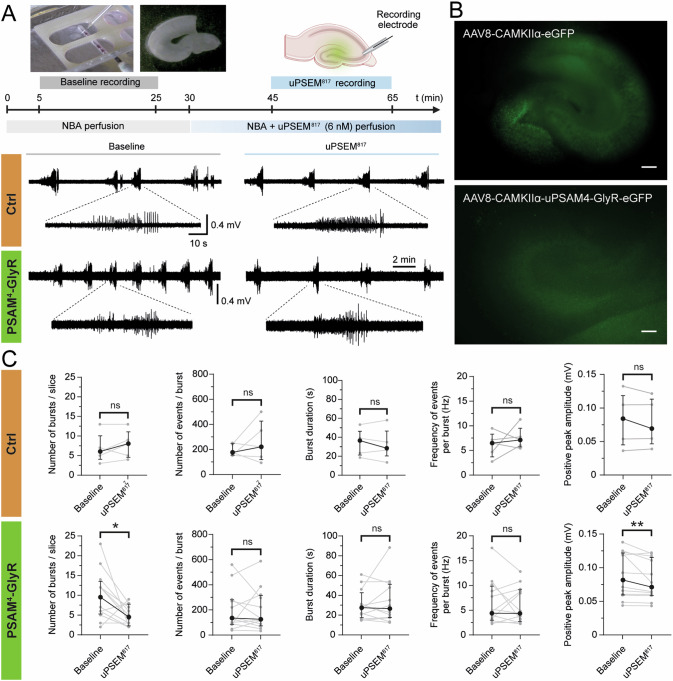
Effect of uPSEM^817^ on epileptic-like activity of entorhinal cortex-hippocampus organotypic slices. **A** Experimental schematic of the timeline to record the epileptiform activity of rhinal cortex-hippocampus organotypic slices at 14 days in vitro (DIV) at baseline condition (grey) and under the effect of 6 nM uPSEM^817^ (blue). Above, example images of the recording set-up, organotypic slices, and the electrode positioning for spontaneous field recordings on CA3. Below, representative traces of epileptic-like activity from slices transduced with AAV8-CaMKIIα-eGFP (orange) and AAV8-CaMKIIα-PSAM^4^-GlyR-IRES-eGFP (green), during baseline (left, grey) and uPSEM^817^ application (right, blue). Burst details are shown in magnified traces. **B** Representative fluorescence images showing GFP expression on the organotypic slices. **C** Characterization of epileptiform-like activity and evaluation of the uPSEM^817^ application effect. From left to right, analysis of the number of bursts per slice, number of events per burst, burst duration, frequency of events per burst, and average positive peak amplitude within each burst. Individual values represent the mean per slice, including *n* = 5 slices transfected with AAV8-CaMKIIα-eGFP (orange) and *n* = 12 slices with AAV8-CaMKIIα-PSAM^4^-GlyR-IRES-eGFP (green). NBA, Neurobasal A medium. Scale bar: 200 µm. Median ± interquartile range. Wilcoxon paired test for comparison of the uPSEM^817^ effect to the baseline. *, *p* < 0.05; **, *p* < 0.01.

Next, we decided to assess the effect of PSAM^4^-GlyR activation by uPSEM^817^ in another epileptic model. We used an ex vivo rat model, the entorhinal cortex-hippocampal organotypic slices that develop spontaneous epileptiform activity after 2 weeks in culture under gradual serum depletion [53, 54]. Three days after culturing, slices were either transfected with control viral vector (AAV8-CaMKIIα-eGFP) or viral vector containing the chemogenetic receptor cassette (AAV8-CaMKIIα-PSAM^4^-GlyR-IRES-eGFP). GFP expression was observed in slices regardless of viral vector. In GFP-only vector slices, GFP fluorescence was present in dentate GCs and some Pyr in CA3 and CA1, although this was not obvious in PSAM^4^-GlyR slices (Fig. 4B). The differential expression can be partially explained by the IRES element driving GFP expression in the PSAM^4^-GlyR vector-treated slices while being directly driven by the CaMKIIα promoter in the control ones. Despite being widely used for co-expression and monitoring gene delivery purposes, IRES-dependent gene expression has been previously shown to be lower [55], and indeed this was observed in slices infected by PSAM^4^-GlyR vector.

At 14 days in vitro, spontaneous activity was assessed by field recordings in the CA3 area. Recordings of 20 min each were analyzed at baseline condition (Fig. 4A, grey) and after the addition of 6 nM uPSEM^817^ (Fig. 4A, blue) to the perfusion media of slices transduced with PSAM^4^-GlyR (*n* = 12) and GFP-only (*n* = 5) vectors. Under baseline conditions, there was no difference in peak amplitude or number of bursts between GFP-only and PSAM^4^-GlyR slices (Supplementary Fig. 1). However, as demonstrated in the representative traces depicted in Fig. 4A, uPSEM^817^ application decreased spontaneous activity compared to the baseline recording in PSAM^4^-GlyR slices (green), but not in GFP-only slices (orange). In PSAM^4^-GlyR slices, perfusion with uPSEM^817^ significantly decreased the number of bursts observed per slice compared to baseline by 47.36% on average (baseline recording: 10.00 ± 1.83 vs uPSEM^817^ recording: 4.92 ± 0.75; *p* = 0.0215; Fig. 4C bottom left, green), while it did not affect slices transfected with GFP-only virus (*p* = 0.375; Fig. 4C top left, orange). Other characteristics of the bursts remained unchanged, on average, in both GFP-only (labeled as Ctrl) and PSAM^4^-GlyR slices (Fig. 4C). This includes the number of events within a burst (*p* = 0.813 GFP-only/Ctrl, and *p* = 0.969 PSAM^4^-GlyR), burst duration (*p* = 0.437 GFP-only/Ctrl, and *p* = 0.850 PSAM^4^-GlyR), and the frequency of events within a burst (*p* = 0.625 GFP-only/Ctrl, and *p* = 0.850 PSAM^4^-GlyR). Lastly, the average positive peak amplitude within each burst was also decreased in the PSAM^4^-GlyR slices (*p* = 0.0093; Fig. 4C bottom right, green), which was not observed in the GFP-only slices (*p* = 0.8125; Fig. 4C top right, orange).

Altogether, these results indicate that the activation of PSAM^4^-GlyR expressed in entorhinal cortex-hippocampus organotypic slices ameliorates epileptiform activity.

### Administration of uPSEM^817^ in PSAM^4^-GlyR transduced chronic epileptic mice did not affect electrographic seizures and IEDs

**Fig. 5:**
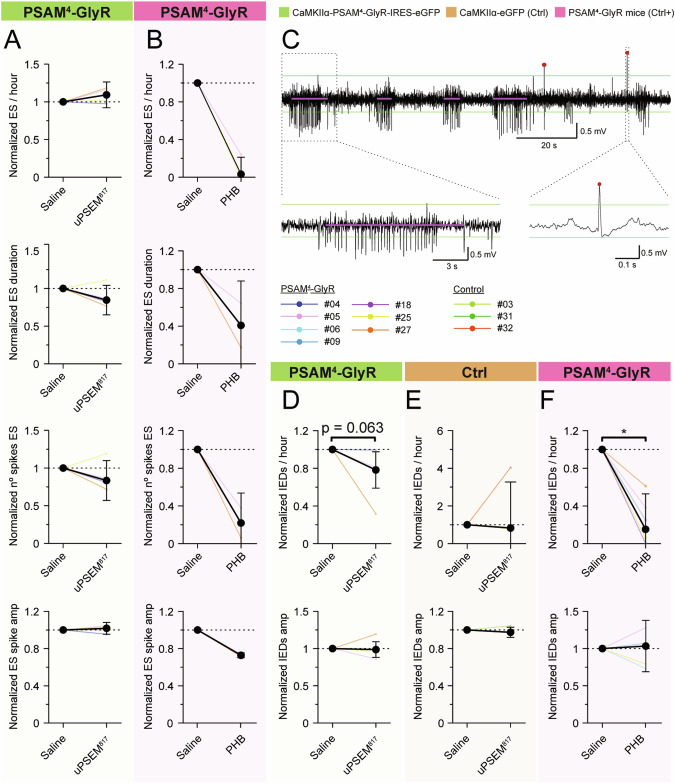
Effect of uPSEM^817^ on ES and IEDs in a chronic model of TLE in mice expressing PSAM^4^-GlyR. **A** Normalized effect of uPSEM^817^ i.p. administration to saline i.p. injection in PSAM^4^-GlyR animals on ES rate, mean ES duration, number of spikes in the ES, and ES spike amplitude (green, *n* = 4). Note that none of the GFP-only vector-treated animals developed spontaneous ES. **B** Normalized effect of PHB in PSAM^4^-GlyR animals on ES rate, mean ES duration, number of spikes in the ES, and ES spike amplitude (magenta, *n* = 3). **C** Example of raw signal with ES detections (magenta line) and IED detections (dark red points). Green lines mark the thresholds for spike detection. Magnification of an ES on the left and of an IED on the right. **D** Normalized effect of uPSEM^817^ i.p. administration in PSAM^4^-GlyR animals on IEDs rate, and amplitude (green, *n* = 5). **E** Normalized effect of uPSEM^817^ i.p. administration on GFP-only vector-treated animals on IEDs rate, and amplitude (orange, *n* = 3). **F** Normalized effect of PHB i.p. administration in both PSAM^4^-GlyR animals on IEDs rate, and amplitude (magenta, *n* = 6). i.p intraperitoneal; PHB phenobarbital; amp amplitude. Median ± interquartile range. Wilcoxon paired test for comparison of the PSEM effect to the saline, and PHB compared to saline.

To investigate whether the activation of PSAM^4^-GlyR by uPSEM^817^ has an effect on ESs in chronic epileptic mice, we analyzed our video-EEG recordings where the animals had received daily intraperitoneal (i.p.) injections of uPSEM^817^, saline (vehicle), or the ASM phenobarbital (see schematic illustration of experimental timeline in Supplementary Fig. 2C). Based on the estimated pharmacokinetics we have chosen to analyze periods of 20–240 min post-injection (see Methods). Administration of uPSEM^817^ i.p. at a dose of 0.03 mg/kg did not affect ES parameters when compared to saline (vehicle) injection in animals transduced by PSAM^4^-GlyR (Fig. 5A and Supplementary Fig. 2A, cyan). In a pair-wise manner (percent change during uPSEM^817^ period normalized to saline period): ES rate (9.3 ± 17%), mean ES duration (−15 ± 20%), mean number of spikes in the ES (−17 ± 26%), or ES spike amplitude (1.8 ± 6.4%). Note that ESs were present in only 4 animals, and therefore may be inconclusive. Phenobarbital (PHB) was used to assess whether a well-established broad-spectrum anti-seizure medication (ASM) acting on GABA_A_ receptors (chloride channels similar to PSAM^4^-GlyR) would be effective under our experimental conditions. Administration of PHB strongly decreased the ES rate (−97 ± 18%), mean ES duration (−59 ± 47%), mean number of spikes in the ES (−78 ± 32%), and ES spike amplitude (−27 ± 2.7%), as seen in Fig. 5B and Supplementary Fig. 2B, magenta. These data confirmed that experimental procedures were valid to allow the detection of seizure-suppressant effects of common ASMs.

We also analyzed the frequency of IEDs, which are pathological high-amplitude spikes in EEG associated with epilepsy [56, 57]. From this analysis, we observed a strong tendency in PSAM^4^-GlyR transduced animals, although not statistically significant, of uPSEM^817^ to decrease the IED rate (−22 ± 19%, *p* = 0.063) without affecting IEDs amplitude (−1.4 ± 11%, *p* = 0.63; Fig. 4D and Supplementary Fig. 2D, cyan). This was not observed in GFP-only animals (IED rate −17 ± 240% and amplitude −2.5 ± 5.5%, *p* = 1, Fig. 5E and Supplementary Fig. 2E, grey). Administration of PHB as a positive control, greatly decreased the IED rate (−85 ± 38%, *p* = 0.031), while it did not affect the IED amplitude (3.3 ± 35%, *p* = 1, Fig. 5F and Supplementary Fig. 2, magenta).

## Discussion

In the present study, we demonstrate that PSAM^4^-GlyR/uPSEM^817^ chemogenetic therapy decreases the excitability of principal neurons in hippocampal slices from epileptic mice. This effect most likely arises from shunting of depolarizing currents in neurons via the activation of transduced chloride-permeable PSAM^4^-GlyR channels by uPSEM^817^, as indicated by a reduction in membrane resistance. Moreover, uPSEM^817^ activation of PSAM^4^-GlyR decreased the number of bursts and peak amplitude of spontaneous epileptiform activity in entorhinal cortex-hippocampal organotypic slices. However, administration of uPSEM^817^ in epileptic animals expressing PSAM^4^-GlyR in the hippocampus did not affect ESs nor IEDs in vivo, although a tendency to decrease the IED rate was observed.

It is well-established that GABA_A_ receptor activation by GABA inhibits neurons by two primary mechanisms: (i) hyperpolarization of cell membrane, as the chloride equilibrium potential is typically more negative than resting membrane potential (RMP), and (ii) by shunting excitatory postsynaptic currents facilitated by the proximity of inhibitory GABAergic synapses to the cell soma and axon hillock [52]. Given that PSAM^4^-GlyR expression occurs across the neuronal membrane, activation by uPSEM^817^ is expected to similarly inhibit AP generation through these mechanisms. Indeed, the initial publication describing the ligand [44] demonstrated neuronal firing suppression in non-epileptic brain tissue due to electrical shunting, evidenced by a reduction in input resistance and an increased current amplitude required to elicit action potentials, among other parameters. Our findings extend this observation to epileptic brain tissue, where we similarly found that larger currents were necessary to induce APs in neurons during uPSEM^817^ application. This supports the notion that the ligand effectively modulates neuronal excitability across both non-epileptic and epileptic contexts.

The role of altered chloride homeostasis in seizure generation across several epilepsy models, including TLE, is a controversial topic [48]. Reports suggest a collapse of transmembrane chloride gradients in epileptic tissue, potentially undermining the hyperpolarizing inhibitory effect mediated by GABA_A_ receptors [58]. Some studies even suggest the activation of GABA_A_ receptors could have depolarizing effects in epileptic patient tissue [49], potentially converting inhibitory neurotransmission into excitatory, thereby increasing neuronal excitability and seizure likelihood. However, other research has not confirmed these changes in chloride homeostasis in epileptic tissue [50, 51, 59]. Our in vitro data support the idea that chloride channel-mediated shunting inhibition can effectively reduce neuronal excitability in chronic epileptic brain slices even without membrane hyperpolarization. Corroborating this, PHB—a barbiturate used as positive control in our in vivo experiments that enhance GABA_A_ receptor activity and increases chloride currents—effectively suppressed ESs and IEDs in epileptic animals. This evidence underscores the potential of chloride channel activation as an anti-seizure strategy in epileptic tissue. Moreover, the use of targeted chemogenetic therapies like PSAM^4^-GlyR/uPSEM^817^ offers significant advantages due to their cell-specificity and a localized impact on the epileptogenic focus. This is in contrast to a traditional well-established broad-spectrum ASM, like PHB, which affects all neurons indiscriminately across the brain, leading to substantial side effects and limiting dose tolerability. Given the challenges of pharmacoresistant epilepsy, future studies should evaluate the PSAM^4^-GlyR/uPSEM^817^ system also in these models, as despite ongoing debates about their reliability, such evaluation is crucial for fully assessing the therapeutic potential of this approach and determining its utility as an alternative or complementary treatment option.

The viral vector-induced expression of PSAM^4^-GlyR in our study was exclusive to excitatory neurons due to the chosen promoter, CaMKIIα, and was mainly restricted to the DG, both at dorsal and ventral hippocampus, although some GFP+ (fluorescent reporter for PSAM^4^-GlyR expression) cells could be found in CA3 and CA1 areas. In our study, coordinates for viral vector injection were designed to target primarily DG, although it is well-established that a limited spread of the viral vector can occur. We used the AAV8 serotype, which may differ in tropism and spread compared to data from other studies using various viral vectors. They include lentiviral particles [59], mixture of rAAV1 and 2 [30, 32], AAV2/7 [40, 60], as well as AAV8 in combination with Cre-recombinase for cell-specific targeting [29]. We have reasoned that targeting our vector into the excitatory cells of the DG should be adequate for controlling seizures. This rationale was grounded in previous studies that have shown reduction in inhibition of GCs following SE [21]. Furthermore, during epileptogenesis newborn dentate GCs exhibit structural and migratory abnormalities which are believed to play a crucial role in development of pro-epileptic neural circuits [39]. These pathophysiological changes in dentate GCs result in aberrant circuit formation characterized by excessive formation of de novo excitatory synapses and recurrent excitatory loops, commonly referred to as mossy fiber sprouting [61]. Normally, the GCs are innately resistant to AP firing and serve as a filter to prevent excessive activation within the hippocampal loop. However, under above mentioned altered conditions, they facilitate the generation, amplification, and propagation of recurrent excitatory signals in the hippocampus [39, 47, 62, 63]. Thus, we hypothesized that enhancing inhibition of DG cells should be sufficient to effectively control chronic seizures. The results of our ex vivo and in vivo studies lend support to this hypothesis, as we observed a significant reduction in bust number and a marked reduction in IED rate in PSAM^4^-GlyR/uPSEM^817^ treated slices and similar trends in animals respectively. On the other hand, these findings also suggest that a broader targeting strategy encompassing additional hippocampal regions might be required for more effective seizure control. Variability in neuronal activity control related to the degree of transduction was previously observed in the original paper describing the PSAM^4^-GlyR system [44]. The authors reported that neurons in densely transduced CA1 hippocampal domains were strongly silenced following uPSEM^792^ i.p. injection, while neurons in the sparsely transduced regions exhibited only modest reductions in activity. Future studies should consider targeting also CA1 and/or CA3 areas of the hippocampus or employing other AAV serotypes that offer wider spread and coverage, potentially enhancing therapeutic outcomes.

Another possible explanation for the minimal in vivo effect of PSAM^4^-GlyR/uPSEM^817^ chemogenetic strategy observed in our study could be a low level of PSAM^4^-GlyR expression in individual neurons. It is conceivable that achieving the maximal therapeutic response might depend on the extent of PSAM^4^-GlyR expression in the excitatory neurons. Additionally, there is a possibility that activation of the PSAM^4^-GlyR channel could lead to an increase chloride load in the cells over time, which in turn could shift the chloride reversal potential towards more depolarized levels of membrane potential, thereby reducing the GABA_A_ receptor-mediated inhibition. However, an effect on the intracellular chloride concentration was not observed in another study using the eGluCl receptor—an LGIC with glutamate-activated chloride pore [59]. In that study, chloride flow through the membrane was transient and spatially restricted, as the receptor was activated only during the seizures when extrasynaptic glutamate levels were elevated.

The pharmacokinetic profile of uPSEM^817^ after intraperitoneal injection in mice has not yet been determined, so we used the data for PSEM^793^ in mice [44] and uPSEM^817^ in rhesus monkeys [64] to estimate the duration of the effect (20–240 min after the injection). If the actual pharmacokinetics of uPSEM^817^ in the mouse differs, we might have missed the period of maximum effect which would render our analysis less sensitive. Using an osmotic pump for continuous delivery of the uPSEM^817^ would be a potential strategy to overcome this issue. A significant effort has been made to modify varenicline, an FDA-approved agonist, to achieve excellent affinity and selectivity for the designer receptor, with uPSEM^817^ emerging as the ligand with the highest affinity (Ki: 0.15 ± 0.02 nM), potency (EC50MP: 0.3 ± 0.4 nM), and exceptional selectivity (5000-fold to 10,000-fold selectivity for PSAM^4^-GlyR over α7-GlyR, α7–5HT3, and 5HT3-R) [44]. However, further improvements to the ligand or the designer receptor could be beneficial, particularly in prolonging the effect of epilepsy treatment. This could reduce the need for multiple daily doses and minimize fluctuations in ligand levels throughout the day.

Gene therapy has previously been shown to decrease neuronal excitability [30, 31, 33]. However, the irreversibility of such interventions presents significant risk that could hinder clinical translation. In contrast, optogenetics and chemogenetics offer more controlled, on-demand approaches, allowing for precise modulation of neuronal network excitability. Optogenetics, while it is powerful and provides millisecond precision, requires light delivery, which adds to the invasiveness of the procedure, posing translational challenges. Chemogenetics, on the other hand, presents fewer barriers to clinical application due to its less invasive nature and controllability offered by an orally administered drug. This allows for dosage adjustments and discontinuation in the event of ineffectiveness or adverse effects on normal brain function. Past studies using chemogenetics [35–40, 60], particularly those employing DREADDs that are G-protein-coupled receptors, have shown promising results in treating drug-resistant epilepsy models. The advantage of DREADDs lies in receptor reserve phenomenon [65], where activation of only a small fraction of receptors is sufficient for maximal downstream effect. Desloovere and colleagues (2019) [40] demonstrated robust suppression of hippocampal seizures for at least 15 h, despite the short half-lives of the agonizts CNO and clozapine (<1 h [66] and about 2 h [67], respectively). This prolonged effect is due to long-term modifications in downstream second-messenger systems, including Gi-protein-coupled inward rectifying potassium channels and reduced neurotransmitter release [66, 68]. However, despite the benefit of prolonged inhibition, a rebound effect has been observed following recovery/withdrawal, which may be relevant to clinical translation [69, 70]. Given the nature of PSAM^4^-GlyR as a modified ion channel, we do not expect prolonged effects beyond the time of agonist availability (reported to be of 3–4 h [44]), as observed in our in vitro measurements after the wash-out period. Nevertheless, the rapid response and the mechanism of action of the PSAM^4^-GlyR/uPSEM^817^ strategy, which mimics well-known ASMs acting on the GABA_A_ receptors, may offer a more translatable therapeutic approach.

The clinical potential of DREADDs may be limited by the pharmacology and off-target effects of the external activator CNO [41, 71]. In this regard, the PSAM^4^-GlyR/uPSEM^817^ system has been suggested as more suitable for clinical settings, particularly for treating drug-resistant epilepsy [72]. Facilitating its translation, varenicline—a molecule already approved for smoking cessation and familiar in clinical settings—serves as the activator, potentially bypassing the extensive safety evaluations required for new drugs [73].

### Concluding remarks

In summary, these findings indicate that although administration of uPSEM^817^ in vitro in slices from PSAM^4^-GlyR transduced epileptic animals inhibits AP generation at a cellular level, it has not proven to be sufficient to suppress epileptic activity in the chronic IHKA mouse model in vivo. The lack of in vivo effect of uPSEM^817^ is likely not due to altered chloride homeostasis but rather inadequate levels and limited spread of PSAM^4^-GlyR expression in the epileptic hippocampus. However, the antiepileptic effect observed on the organotypic slices is encouraging. This suggests that while the PSAM^4^-GlyR/uPSEM^817^ strategy effectively suppresses neuronal excitability, further optimization is required to achieve a therapeutic impact in epileptic animals and potentially in humans in the future.

## Material and methods

### Animals

Experiments were conducted on adult (2–3.5 months of age) wild-type C57BL/6J male mice (Janvier Labs). Mice were housed at the local animal facility, under 12 h light/12 h dark cycle with access to food and water ad libitum, and in individually ventilated cages. Three cohorts of animals were used: cohort 1, *n* = 6; cohort 2, *n* = 10; cohort 3, *n* = 15 (total *n* = 31). A maximum of 5 mice was kept in each cage, mice were tagged with an earmark number and randomly assigned to experimental groups. After viral injections, 3 mice died (total *n* = 28; PSAM *n* = 21; GFP-only/Ctrl *n* = 7), and during status epilepticus induction 10 mice died (total *n* = 18; PSAM *n* = 14; GFP-only/Ctrl *n* = 4), leaving a total of 18 mice video-EEG monitored. EEG signal had insufficient quality for analysis in 5 animals (PSAM *n* = 3; GFP-only/Ctrl *n* = 2), and 4 animals did not develop SRS nor ESs, leaving a total of 9 animals used for analysis (total *n* = 9; PSAM *n* = 7; GFP-only/Ctrl *n* = 2).

The experimental procedures performed were approved by the Malmö/Lund Animal Research Ethics Board, ethical permit number 2998/2020-m, and conducted in agreement with the Swedish Animal Welfare Agency regulations and the EU Directive 2010/63/EU for animal experiments.

Organotypic slices were prepared using Sprague-Dawley rats at Instituto de Medicina Molecular João Lobo Antunes (iMM). All experiments were conducted according to European Union Guidelines (2012/707/EU) and to the Portuguese legislative action (DL 113/2013) for the protection of animals used for scientific purposes. The methods described were approved by “iMM’s Institutional Animal Welfare Body (ORBEA-iMM, Lisboa, Portugal) and authorized by the Portuguese authority for Animal Welfare (Direção Geral de Alimentação e Veterinária—DGAV).

### Virus production and injection

AAVs were produced as previously described [74]. In brief, AAV8-CaMKIIα-PSAM^4^-GlyR-IRES-eGFP (was a gift from Scott Sternson; Addgene plasmid # 119744; http://n2t.net/addgene:119744; RRID: Addgene_119744) and AAV8-CaMKIIα-GFP genomes were packaged separately into AAV8 via PEI transfection. HEK293T cells were double transfected with AAV8-CaMKIIα-PSAM^4^-GlyR-IRES-eGFP and pDP8.ape (PlasmidFactory GmbH & Co. KG; # PF478). AAVs were harvested 72 h post-transfection using polyethylene glycol 8000 (PEG8000) precipitation and chloroform extraction, followed by PBS exchange in concentration columns. Purified AAVs were titered using RT-qPCR with standard curves and primers specific for the ITRs. AAVs were stored in glass vials at 4 °C until use. Titers used were around 8 × 10^13^ gc/ml, and if needed were normalized using PBS.

Intrahippocampal injections were performed via stereotaxic surgery. In brief, mice were anesthetized with isoflurane/O_2_ mixture 5% and placed in the stereotaxic frame, thereafter anesthesia was maintained at 1.5%. After bupivacaine injection (<0.5 ml, Marcaine, AstraZeneca) a middle incision was made on top of the skull. Small holes were drilled at the injection sites and bilateral injections were targeted to the following coordinates (in mm, from Bregma): position 1: AP −2.2, ML ± 1.7, DV −1.9; position 2: AP −3.3, ML ± 3.0, DV −3.7 and −2.7. A total volume of 0.4 µl was injected at a speed of 0.1 μl/min with an additional 3 min allowed for diffusion before careful retraction of the needle and moving to the next coordinate. A total of 1.2 µl was injected in each hemisphere using a glass capillary and injection pump (Nanoliter 2010, World Precision Instruments). The wound was sutured with resorbing thread (Vicryl, Ethicon).

### Kainic acid-induction of status epilepticus in adult mice and electrode implantation

IHKA was chosen as a well-established mouse model of chronic TLE with HS. This model is used by the NIH/NINDS Epilepsy Therapy Screening Program to test the efficacy of new antiepileptic treatments since it mimics drug-resistant seizures as described in TLE patients [75].

A minimum of two weeks after virus injection, mice were again anesthetized and placed in the stereotaxic frame as described above. Unilateral KA injections were performed at the right dorsal hippocampus following coordinates (in mm, from Bregma): AP −2.0, ML + 1.6, DV −1.9. A total of 45 nl of KA solution (20 mM i.e., total dose 0.9 nM KA; Abcam ab120100) was injected at a rate of 25 nl/min followed by 3 min of waiting time before retraction of the glass capillary.

During the same surgery, the electrode (E363/2, P1 Technologies) and, after blunt dissection creating a skin pocket over the right dorsal thoracic region, a telemetry transmitter (MT10B, KAHA Sciences) for EEG recording were implanted. In brief, an insulated stainless-steel electrode (203 µm diameter) was inserted at the same coordinates as for KA injection. The positive lead of the transmitter was connected before insertion by clamping to the electrode connector, and sealed with conductive paint (Bare Conductive). The negative (reference) lead was similarly connected to a skull screw (AgnTho’s) in the occipital or parietal bone. Connections were fixed on the skull and insulated with first cyanoacrylate glue (Super Glue, Loctite) followed by dental acrylic cement (AgnTho’s) and the opening of the skin pocket containing the transmitter was sutured with resorbing thread.

In our study, the IHKA mouse model deviated from the previously described model in the literature [75–78], complicating the assessment of the therapeutic outcomes. Typically, the majority of IHKA-treated mice exhibit numerous focal nonconvulsive electrographic seizures, with behavioral seizures being infrequent. However, in the current study, 4 out of 9 IHKA mice developed electrographic seizures exclusively, while all displayed frequent behavioral seizures. This divergence from prior studies could be attributed to the concurrent IHKA injections and electrode implantation during the same surgical session, potentially causing a greater spread of the kainic acid (KA) within the hippocampus, including along the electrode track. This likely resulted in more extensive tissue damage and disruption of the BBB, leading to a more severe epileptic phenotype. Future experimental designs should take into account the potential increased KA spread and BBB disruption when combining procedures. Additionally, the experimental design might need to address the issue of neuronal loss following SE induction post-viral vector injection, which could reduce the effectiveness of the uPSEM^817^ treatment.

### Intrahippocampal EEG recordings and drug treatment

The EEG was recorded using a telemetry system (MT110 tBase, Kaha Sciences) connected to an ADC (PowerLab, AD Instruments) with LabChart Pro software (AD Instruments) on a Windows PC. The sampling rate was 1 kHz. The video was registered using two HD ethernet cameras (Axis Communications), combined in the open-source Open Broadcaster Software (OBS Studio), and synchronized and recorded using LabChart. After the surgery, the animals were video-EEG monitored for status epilepticus for at least 24 h before being returned to stables. Two weeks later, continuous video-EEG monitoring commenced during which the different treatment conditions were administered according to the schedule (Supplementary Fig. 2C): uPSEM^817^ (PSAM^4^ ligand), saline (vehicle and negative control treatment), and PHB (ASM, positive control treatment).

All treatments were administered at the same time of the day (10:30 a.m.) to avoid possible circadian influence. uPSEM^817^ diluted in saline at the concentration of 0.05 mg/ml was administered i.p. at the dose 0.03 mg/kg [44]. As a negative control treatment, the corresponding volume of saline was administered i.p. As a positive control for uPSEM^817^, we injected i.p. PHB at the dose of 40 mg/kg [51]. After the end of the in vivo experiments, the animals were either perfused or used for in vitro electrophysiological experiments.

### EEG data analysis

The in vivo data were analyzed using LabChart software and Matlab R2019b. LabChart was used to manually review the recordings and label treatment administration and large behavioral seizures (LBSs) [79]. The behavioral severity was quantified using the Racine scale [80] with added sixth class representing wild running seizure type. For loading the signals and labels from the LabChart *.adicht files into the Matlab environment we wrote our scripts and utilized ADInstruments (LabChart) SDK [81].

For the detection of ESs and IEDs, we used a semi-automatic Matlab-based Electrographic seizure analyzer (ESA) available at https://github.com/KM-Lab/Electrographic-Seizure-Analyzer [79]. The thresholds for the detection of spikes and artifacts were set individually for each animal using the plotting function of ESA to maximize the number of true detections while not allowing false detections. We used the default settings of the ESA except for the ES detection which we required to have at least 8 spikes in each 4 s. The minimum seizure length was 4 s and ESs closer than 4 s were glued together. For the ES detection, we used negative spikes. In the animals in which ESs were present, we considered only positive spikes to be IEDs. In the animals which did not present ESs, we have chosen the polarity of spikes which was more frequent to be considered as IEDs. We considered only narrow spikes as IEDs (Fig. 4A).

For the ESs we analyzed the following five parameters: ES rate, mean ES duration, mean number of spikes constituting the ES, and the mean spike amplitude within the ES. For the IEDs, we determine their rate and mean amplitude. In the calculation of the ES rate, we excluded the time spent in behavioral seizures or the artifact-contaminated periods. To determine the IED rate, we also excluded the time spent in the ESs.

The effect of uPSEM^817^ was analyzed from 20 min to 240 min after i.p. injection, to avoid any possible injection effect contamination and due to previous data in PSEM pharmacokinetics [44, 64]. For PHB analysis data from 10 min to 70 min after i.p. injection was considered following the previous reasoning [51]. Data are represented as the median ± interquartile range of the median fold change of the subjects after normalization to saline injection. Importantly, for the GFP-only group, no animals displayed ESs, and for IED analysis #32 corresponds to a repeated recording in animal #31.

### In vitro electrophysiology

Whole-cell patch-clamp recordings were performed in acute hippocampal slices. First, mice, after being removed from the video-EEG monitoring, were briefly anesthetized with isoflurane and decapitated. Brains were transferred to an ice-cold sucrose-based cutting solution containing (in mM): 75 sucrose, 67 NaCl, 26 NaHCO_3_, 25 D-glucose, 2.5 KCl, 1.25 NaH_2_PO_4_, 0.5 CaCl_2_, 7 MgCl_2_ (all from Sigma Aldrich), equilibrated with carbogen (95% O_2_/5% CO_2_), with pH 7.4 and osmolarity ~305–310 mOsm. Hemispheres of the brains were separated and cut on a vibratome (VT1200S, Leica Microsystems) into 400 μm thick slices. The right hemisphere was cut sagittal to better visualize the dorsal hippocampus where the electrode was implanted, and the left hemisphere was cut quasi-horizontal [82]. Slices were incubated in this cutting solution for 30 min at 34 °C, and subsequently transferred to aCSF containing (in mM): 119 NaCl, 26 NaHCO_3,_ 25 D-glucose, 2.5 KCl, 1.25 NaH2PO_4_, 2.5 CaCl_2_ and 1.3 MgCl_2_ (pH 7.4, osmolarity 305–310 mOsm). Slices were kept in aCSF at room temperature (RT) until recordings were performed. The individual cells in the slices were visualized for whole-cell patch-clamp recordings using infrared differential interference contrast video microscopy (BX51WI; Olympus). Recordings were performed from GFP+ cells (identified under fluorescent 470 nm light) at 32 °C using a glass pipette filled with a solution containing (in mM): 140 K-Gluconate, 4 NaCl, 10 KOH-HEPES, 0.2 KOH-EGTA, 2 Mg-ATP, and 0.3 Na_3_GTP (~300 mOsm, pH 7.2; all from Sigma-Aldrich). The average pipette tip resistance was between 2.5 and 6 MΩ. Pipette capacitance was corrected online before GΩ seal formation while fast capacitive currents were compensated for during cell-attached configuration. All recordings were done using a HEKA EPC9 amplifier (HEKA Elektronik) and sampled at 10 kHz with a 3 kHz Bessel anti-aliasing filter and using PatchMaster software for data acquisition. For each recording, baseline parameters were measured immediately after accessing the cell and again 3–5 min later, just before initiating drug wash-in. The measurements taken just prior to the application were used for analysis. uPSEM^817^ was then introduced at a final concentration of 3 nM, dissolved in aCSF, using a perfusion pump. The effects of uPSEM^817^ were assessed 30–40 min after the start of the wash-in. Finally, 30–40 min after beginning the wash-out period, during which regular aCSF without the drug was perfused, measurements for the wash-out parameters were taken.

Passive membrane properties of GFP+ cells and spontaneous synaptic activity: After the formation of a GΩ seal, the patch was ruptured giving direct access to the intracellular compartment. RMP was determined in current-clamp mode at 0 pA immediately after establishing the whole-cell configuration. Series resistance (Rs) and input resistance (Ri) were determined in voltage clamp at −70 mV from a 5 mV negative voltage pulse applied through the patch pipette and monitored throughout the experiment. A series of square current steps of 500 ms duration from −40 pA to 200 pA in 10 pA steps, were applied at a membrane potential of approximately −70 mV with holding current as needed, to determine the cells’ ability to generate AP. AP characteristics were assessed by administration of a depolarizing ramp current over 1 s, from a holding potential of −70 mV, starting with a 0–25 pA ramp and up to a 0–500 pA ramp in various cells. Spontaneous postsynaptic potentials were recorded at 0 pA holding current.

### Preparation of rhinal cortex-hippocampus organotypic slices

Rhinal cortex-hippocampus organotypic slices were prepared from 6–7 days-old Sprague-Dawley rats, according to previous reports [47, 48]. No sex determination was conducted on the pups. Each experiment utilized the entire litter, which included both male and female pups. Rats were euthanized by decapitation and the brain removed, under sterile conditions, to a 60 mm plate with ice-cold Gey’s balanced salt solution (GBSS) (Biological Industries, Israel) supplemented with 25 mM D-glucose (Sigma, USA). The hippocampus, entorhinal cortex, and perirhinal cortex were separated and sliced transversely (350 µm thick) using a McIlwain tissue chopper. Four slices were transferred to porous (0.4 µm) insert membranes (PICM 03050, Millipore, USA) placed in wells of six-well culture trays (Corning Costar, Corning, USA) containing 1.1 mL of culture medium composed of 50% Opti-MEM, 25% Hanks’ balanced salt solution (HBSS), 25% heat-inactivated horse serum (HS), 30 μg/mL gentamycin (Thermo Fisher, USA), and 25 mM D-glucose (Sigma). Slices were maintained at 37 °C with 5% CO_2_ and 95% O_2_ for the following 2 weeks. From 3 days in vitro (DIV) on, slices were changed to supplemented Neurobasal A (NBA) medium (NBA, 2% B-27, 1 mM L-glutamine, gentamycin 30 μg/mL, all from Thermo Fisher) and subjected to decreasing HS concentrations (15%, 10% and 5%) until a serum-free medium was reached. Culture medium was changed every other day.

At 3 DIV, 2 µl of either AAV8-CAMKIIα-eGFP or AAV8-CAMKIIα-uPSAM4-GlyR-IRES-eGFP viral vector, diluted 1:10 in NBA, was carefully placed on the top of the hippocampus. Just before recording, each slice was visually inspected, to ensure slice integrity, and a fluorescence image was acquired in a Zeiss Axiovert 200 fluorescence microscope, equipped with an AxioCam MRm, using the AxioVision imaging software (Zeiss, Germany).

### Spontaneous activity recordings from organotypic slices

Under a gradual deprivation of serum rhinal cortex-hippocampus organotypic slices depict spontaneous epileptiform discharges [47, 48]. At 14 DIV individual slices were transferred to an interface-type chamber with a humidified (5% CO_2_/95% O_2_) atmosphere at 37 °C, and with the NBA medium continuously recirculating at a rate of 2 mL/min. After a stabilization period of 5 min, the baseline epileptiform activity was recorded for 20 min. The superfused medium was then changed to NBA supplemented with 6 nM uPSEM^817^. After an equilibration period of 15 min, to ensure a complete renewal of medium bathing the slice, a 20 min recording was acquired (Recording scheme in Fig. 4A). Spontaneous field potentials were recorded using a glass micropipette electrode (2–4 MΩ) filled with artificial cerebrospinal fluid (aCSF) containing (in mM): 124 NaCl, 3 KCl, 1.2 NaH_2_PO_4_, 25 NaHCO_3_, 10 glucose, 2 CaCl_2_ and 1 MgSO_4_ (pH 7.4), and positioned in the CA3 pyramidal cell layer. Recordings were obtained with an Axoclamp 2B amplifier (Axon Instruments, USA), and digitized with the WinLTP software (WinLTP Ltd., UK) [83].

In this study, ictal-like discharges were defined as continuous discharges lasting more than 10 s (bursts) or with a minimum frequency of 2 Hz, and the end of a burst was defined when the inter-spike interval was longer than 2 s [84]. Continuous activity that did not fit within these parameters was not considered burst activity. The pCLAMP Software Version 10.7 (Molecular Devices Corporation, California, USA) was used to automatically detect the events during a recording. All recordings were band-pass filtered (eight-pole Bessel filter at 60 Hz and Gaussian filter at 600 Hz).

The baseline used in pCLAMP to detect the events was specific to each recording and was established right above the noise of oscillations. The number of bursts per slice, the number of events within a burst, and the duration of each burst, as well as the frequency of events and the average positive peak amplitude (amplitude between the baseline and the peak of the spike) in a burst, were automatically evaluated in an in-house program developed in C^++^ language.

### Immunofluorescence

Hippocampal slices were fixed after acute recordings overnight at 4 °C with 4% PFA and changed to KPBS after. Then, slices were washed thoroughly three times with KPBS, incubated for 1 h at RT in permeabilization solution (0.02% BSA  + 1% Triton X‐100 in PBS) and 2 h at RT in blocking solution (5% normal serum +1% BSA + 0.2% Triton X‐100 in PBS). Primary antibodies (Chicken anti-GFP, 1:400, Abcam ab13970) were diluted in blocking solution and incubated for 48 h at 4 °C. Then, slices were incubated again in blocking solution for 2 h at RT. Secondary antibodies (AlexaFluor Plus 488 Goat anti-chicken, 1:1000, ThermoFisher) were applied in blocking solution for 48 h at 4 °C. Nuclei were counterstained with Hoechst 33342 (1:1000) diluted in the last rinsing with PBS for 20 min before mounting with PVA-DABCO mounting media. Images were acquired by confocal microscopy (Nikon Confocal A1RHD microscope).

For mossy fiber sprouting and dentate GC layer dispersion, slices were subcutted in the microtome to 30 μm thickness. Then, sections were washed thoroughly three times with KPBS and then pre-incubated for 1 h in blocking solution containing 10% normal serum (of the species-specific to the secondary antibody) in KPBS containing 0.25% Triton-X-100, for 1 h at RT. Primary antibodies diluted in the blocking solution were incubated overnight at 4 °C (Rabbit anti-ZnT3 [Synaptic systems 197003, 1:500], and mouse anti-NeuN [Millipore MAB377, 1:400]). Following primary antibody incubation, sections were washed three times with KPBS and incubated again in blocking solution for 1 h at RT. Then, sections were incubated with secondary antibodies for 2 h at RT (AlexaFluor Plus 555 goat anti-rabbit or mouse, 1:1000, ThermoFisher). Nuclei were counterstained with Hoechst 33342 (1:1000) diluted in the last rinsing with PBS before mounting with PVA-DABCO mounting media. Images were acquired by epifluorescence microscopy (Olympus BX61 and Leica DMi8).

### Statistical analyses

Whole-cell patch-clamp recordings were analyzed offline with Igor Pro (Wavemetrics). The cell was excluded from analysis if Rs changed during the recording by more than 20%. On-ramp recordings, AP amplitude was measured from threshold to peak and the minimum current needed for the first AP was measured. On step recordings, AP amplitude and threshold were measured as before, and the amplitude of the afterhyperpolarization (AHP) was measured as the difference between the AHP peak and the AP threshold. For the 500 pA steps, the steady-state amplitude was measured as the difference between the holding potential, −70 mV, and the potential at the plateau of the pulse response.

For fluorescence quantification, an image of the right dorsal DG, at the level of the electrode placement was digitally acquired using the 10X objective (epifluorescence microscopy, Olympus BX61). All images were taken with the same exposure time and ISO and treated equally. At least three different hippocampal sections were used for the analysis of each animal. Subsequently mean fluorescence intensity was measured using Fiji/ImageJ software (NIH, Annapolis, MD, USA). DG area was outlined and the mean gray value was measured within the selected area. Moreover, the maximum gray value was also measured for each area.

Statistical analysis of the data was performed using Prism 7 (GraphPad). Data were represented as paired, and medians ± interquartile range was used for group representation. Wilcoxon rank test was used for comparison of medians with Bonferroni’s *post hoc* test for multiple comparisons of medians. Statistical analysis between the activity parameters evaluated in the baseline recording of each organotypic slice and the ones assessed in the recording under uPSEM^817^ superfusion was achieved by a paired Wilcoxon test. The level of significance for the tests was set at *p* < 0.05. All data is presented in the figures as medians ± interquartile range.

## Supplementary information

Supplemental Figure legends

Supplementary Table 1

Supplementary Figure 1

Supplementary Figure 2

## Data Availability

The data generated and analyzed during this study can be found within the published article and its supplementary files.
