# Supplementary material for: Chemogenetics with PSAM4-GlyR decreases excitability and epileptiform activity in epileptic hippocampus
Source: Gene Ther. 2024 Oct 25;32(2):106–20. doi: 10.1038/s41434-024-00493-7 (PMC11946892; doi:10.1038/s41434-024-00493-7)
Supplement: Supplementary file 1 — Supplemental Figure legends [file 41434_2024_493_MOESM1_ESM.docx]

**Supplemental information**

**Tables**

**Supplementary Table 1. Intrinsic electrophysiological properties of the cells in acute brain slices.** Changes in intrinsic properties of the cells recorded at baseline, during application of uPSEM^817^ 3 nM, and after washout. n, number of cells; Rs, series resistance; Ri, input resistance; AP, action potential; APth, AP threshold; APImin, the minimum current needed for the first AP; APamp, amplitude; APahp, afterhyperpolarization amplitude; Step 500, 500 pA depolarizing step current. (^#^) Normalized values to baseline levels. Median [interquartile range]. Wilcoxon paired test. *, p < 0.05; **, p < 0.01; ns, not significant.

**Supplementary Figure Legends**

**Supplementary Figure 1. Comparison of epileptic-like activity parameters and IED rate during baseline recordings.** The two graphs on the left refer to the organotypic slice recordings. Positive peak amplitude (left) and number of bursts per slice (right) during baseline recording in both GFP-only (Ctrl) and PSAM^4^-GlyR transduced conditions. The graph on the right illustrates in vivo EEG data, Showing the IEDs per hour in GFP-only (Ctrl) and PSAM4-GlyR virus-injected mice. The analyses focus on the time periods from 10:30 until 14:30 of each baseline day (consistent with the intervals used for assessing treatment efficacy). Median ± interquartile range. Wilcoxon test.

**Supplementary Figure 2. Effect of uPSEM^817^ on ES and IEDs in epileptic mice expressing PSAM^4^-GlyR. (A)** Effect on ES rate, mean ES duration, number of spikes in the ES, and ES spike amplitude, of uPSEM^817^ i.p. administration compared to saline i.p. injection in PSAM^4^-GlyR transduced animals (green, n = 4). **(B)** Effect of PHB on ES rate, mean ES duration, number of spikes in the ES, and ES spike amplitude in PSAM^4^-GlyR transduced animals (magenta, n = 3). **(C)** Schematic illustration of the timeline for the different treatments applied to mice *in vivo*. **(D)** Effect on IEDs rate, and amplitude of uPSEM^817^ i.p. administration in PSAM^4^-GlyR animals (green, n = 5). **(E)** Effect uPSEM^817^ i.p. administration on IEDs rate, and amplitude in control animals (orange, n = 3). **(F)** Effect of PHB i.p. administration on IEDs rate, and amplitude in PSAM^4^-GlyR animals (magenta, n = 6). I.p., intraperitoneal; PHB, phenobarbital; amp, amplitude. Median ± interquartile range. Wilcoxon paired test for comparison of the PSEM effect compared to the saline, and PHB compared to saline.
