# Supplementary material for: Chemogenetics with PSAM4-GlyR decreases excitability and epileptiform activity in epileptic hippocampus
Source: Gene Ther. 2024 Oct 25;32(2):106–20. doi: 10.1038/s41434-024-00493-7 (PMC11946892; doi:10.1038/s41434-024-00493-7)
Supplement: Supplementary file 2 — Supplementary Table 1 [file 41434_2024_493_MOESM2_ESM.docx]

**Supplementary Table 1. Intrinsic electrophysiological properties of the cells in acute brain slices.**

Changes in intrinsic properties of the cells recorded at baseline, during application of uPSEM817 3 nM, and after washout. n, number of cells; Rs, series resistance; Ri, input resistance; AP, action potential; APth, AP threshold; APImin, the minimum current needed for the first AP; APamp, amplitude; APahp, afterhyperpolarization amplitude; Step 500, 500 pA depolarizing step current. (#) Normalized values to baseline levels. Median [interquartile range]. Wilcoxon paired test. *, *p* < 0.05; **, *p* < 0.01; *ns*, not significant.

|  | **PSAM^4^-GlyR-IRES-eGFP+ cells** | | | **eGFP+ cells (control)** | | |
| --- | --- | --- | --- | --- | --- | --- |
|  | **Baseline** | **uPSEM^817^** | **Wash out** | **Baseline** | **uPSEM^817^** | **Wash out** |
| **n** | 12 | 12 | 7 | 7 | 7 | 2 |
| **Rs** (MΩ) | 29.6  [23.93, 62.48] | 36 **ns**  [24.15, 54.95] | 42.7  [27.1, 66.03] | 35.8  [26.5, 50.9] | 37.6 **ns**  [28, 55] | 41.1  [33.6, 48.6] |
| **Ri** (MΩ) | 381.5  [297.8, 603.5] | 349 ******  [245.3, 372.3] | 262  [227, 421] | 265  [167, 376] | 221 **ns**  [196, 407] | 192  [171, 213] |
| **Ri ^#^** | 1 | 0.7847 ******  [0.617, 0.869] | 0.7992  [0.740, 0.865] | 1 | 1.082 **ns**  [0.822, 1.257] | 0.937  [0.792, 1.082] |
| **Ramp** |  |  |  |  |  |  |
| n AP | 6  [4, 11] | 4.5 ******  [1, 7.75] | 5  [4, 12] | 5  [3.25, 12.75] | 7.5 **ns**  [2.5, 16.25] | 4  [2, 6] |
| n AP ^#^ | 1 | 0.55 ******  [0.417, 0.906] | 1  [0.854, 1.031] | 1 | 1.167 **ns**  [0.813, 1.688] | 1.75  [1.5, 2.0] |
| AP_th_ (mV) | -30.94  [-23.29, -33.38] | -31.5 **ns**  [-26.04, -35.08] | -33.47  [-22.7, -38.74] | -29.15  [-27.67, -32.9] | -31.03 **ns**  [-30.53, -37.72] | -28.77  [-27.25, -30.3] |
| AP_Imin_ (pA) | 59.76  [47.95, 90.51] | 86.37 ******  [57.55, 110.3] | 60.38  [31.59, 102.3] | 158.9  [113.6, 241.6] | 121 **ns**  [101, 226.3] | 230.3  [203.8, 256.9] |
| AP_Imin_ ^#^ | 1 | 1.19 ******  [1.048, 1.329] | 1.026  [0.94, 1.071] | 1 | 0.844 **ns**  [0.766, 1.079] | 0.951  [0.917, 0.984] |
| AP_amp_(mV) | 84.4  [72.02, 91.44] | 76.81 ******  [68.7, 90.41] | 77.59  [45.15, 90.19] | 83.62  [69.98, 96.64] | 86.45 **ns**  [73.66, 96.11] | 76.48  [68.28, 84.68] |
| **Step I_min_** |  |  |  |  |  |  |
| n AP | 3.5  [2, 5] | 1.5 **ns**  [1, 2.25] | 2  [0, 4] | 1  [0.5, 2] | 2 **ns**  [1, 3.5] | 1  [1, 1] |
| n AP ^#^ | 1 | 0.5 **ns**  [0.25, 0.625] | 0.25  [0, 1.625] | 1 | 2.5 **ns**  [1, 3.75] | 0.5  [0, 1] |
| I_min_ (pA) | 80  [45, 100] | 85 **ns**  [50, 132] | 110  [70, 180] | 130  [75, 177.5] | 100 **ns**  [75, 147.5] | 170  [160, 180] |
| AP_amp_(mV) | 79.22  [68.91, 89.88] | 73.37 *****  [56.10, 85.55] | 79.81  [70.75, 88.87] | 84.25  [73.34, 95.78] | 86.21 **ns**  [70.54, 95.35] | 78.51  [71.06, 85.96] |
| AP_ahp_(mV) | 11.33  [7.898, 17.58] | 10.06 **ns**  [6.458, 18.05] | 11.29  [7.65, 14.93] | 17.41  [12.02, 19.36] | 17.8 **ns**  [8.2, 19.46] | 15.62  [12.12, 19.12] |
| **Step 500** |  |  |  |  |  |  |
| n AP | 12  [6, 28] | 9 ******  [3, 25] | 5.5  [2.75, 26.75] | 13  [10, 20] | 15 **ns**  [11, 24] | 12.5  [8, 17] |
| n AP ^#^ | 1 | 0.75 ******  [0.5, 0.8929] | 0.33  [0, 0.8571] | 1 | 1.25 **ns**  [0.93, 1.3] | 0  [0, 1.214] |
| Steady state (mV) | 62.62  [52.15, 73.58] | 61.01 *****  [41.23, 68.97] | 62.87  [38.48, 72.03] | 58.62  [54.83, 80.56] | 63.28 **ns**  [54.87, 69.59] |  |
