# Supplementary figures and images for: Chemogenetics with PSAM4-GlyR decreases excitability and epileptiform activity in epileptic hippocampus
Source: Gene Ther. 2024 Oct 25;32(2):106–20. doi: 10.1038/s41434-024-00493-7 (PMC11946892; doi:10.1038/s41434-024-00493-7)

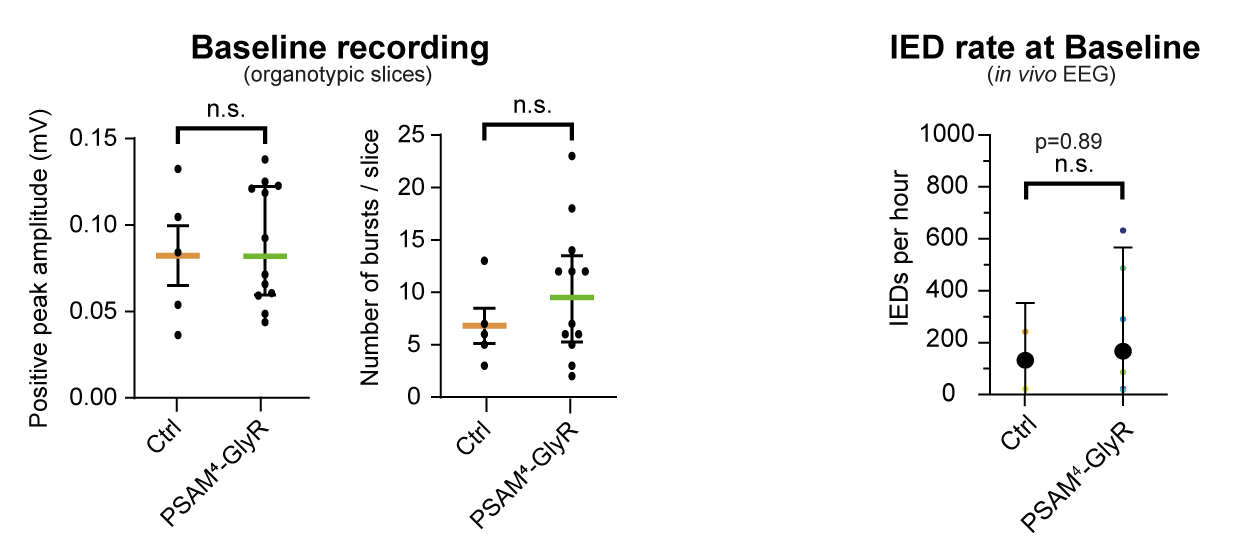

Supplement: Supplementary file 3 — Supplementary Figure 1 [file 41434_2024_493_MOESM3_ESM.tif]

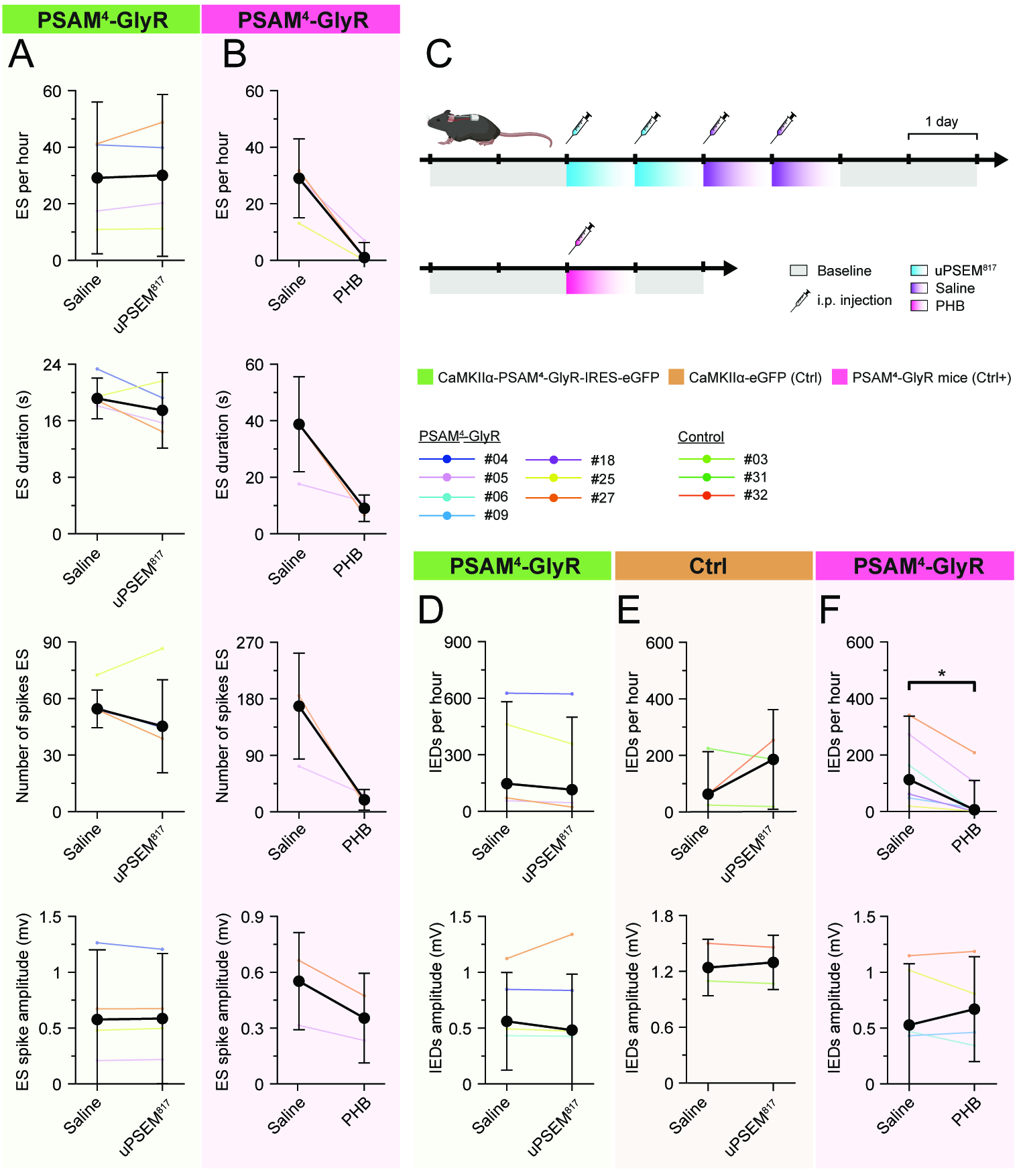

Supplement: Supplementary file 4 — Supplementary Figure 2 [file 41434_2024_493_MOESM4_ESM.tif]
